# Mapping of NKp46^+^ Cells in Healthy Human Lymphoid and Non-Lymphoid Tissues

**DOI:** 10.3389/fimmu.2012.00344

**Published:** 2012-11-20

**Authors:** Elena Tomasello, Nadia Yessaad, Emilie Gregoire, Kelly Hudspeth, Carmelo Luci, Domenico Mavilio, Jean Hardwigsen, Eric Vivier

**Affiliations:** ^1^Centre d’Immunologie de Marseille-Luminy, Aix-Marseille Université UM2Marseille, France; ^2^Institut National de la Santé et de la Recherche Medicale, UMR 1104Marseille, France; ^3^Centre National de la Recherche Scientifique, Unite Mixte de Recherche 7280Marseille, France; ^4^Assistance Publique des Hôpitaux de Marseille, Hôpital de la ConceptionMarseille, France; ^5^Unit of Clinical and Experimental Immunology, Humanitas Clinical and Research CenterMilan, Italy; ^6^Department of Medical Biotechnologies and Translational Medicine, University of MilanMilan, Italy; ^7^CNRS UMR 7275, Institut de Pharmacologie Moléculaire et CellulaireValbonne-Sophia Antipolis, France

**Keywords:** NKp46, human NK cells, tissue distribution, phenotype, expression profiling

## Abstract

Understanding Natural Killer (NK) cell anatomical distribution is key to dissect the role of these unconventional lymphocytes in physiological and disease conditions. In mouse, NK cells have been detected in various lymphoid and non-lymphoid organs, while in humans the current knowledge of NK cell distribution at steady state is mainly restricted to lymphoid tissues. The translation to humans of findings obtained in mice is facilitated by the identification of NK cell markers conserved between these two species. The Natural Cytotoxicity Receptor (NCR) NKp46 is a marker of the NK cell lineage evolutionary conserved in mammals. In mice, NKp46 is also present on rare T cell subsets and on a subset of gut Innate Lymphoid Cells (ILCs) expressing the retinoic acid receptor-related orphan receptor γt (RORγt) transcription factor. Here, we documented the distribution and the phenotype of human NKp46^+^ cells in lymphoid and non-lymphoid tissues isolated from healthy donors. Human NKp46^+^ cells were found in splenic red pulp, in lymph nodes, in lungs, and gut lamina propria, thus mirroring mouse NKp46^+^ cell distribution. We also identified a novel cell subset of CD56^dim^NKp46^low^ cells that includes RORγt^+^ ILCs with a lineage^−^CD94^−^CD117^bright^CD127^bright^ phenotype. The use of NKp46 thus contributes to establish the basis for analyzing quantitative and qualitative changes of NK cell and ILC subsets in human diseases.

## Introduction

Originally assigned to the innate immune system, Natural Killer (NK) cells recently revealed adaptive properties (Paust and von Andrian, 2011; Sun and Lanier, 2011; Vivier et al., 2011). Beside their unique ability to exert natural and antibody-dependent cell cytotoxicity (ADCC), NK cells secrete various cytokines and chemokines, thus shaping both innate and adaptive immune responses (Vivier et al., 2008). Consistent with their patrolling role during early immune responses, NK cells are broadly distributed in the mouse in both lymphoid (e.g., peripheral blood, lymph nodes, spleen, and bone marrow) and non-lymphoid organs (e.g., liver, lung, uterus, skin, and gut; Shi et al., 2011). Distinct combinations of homing and chemotactic G-protein coupled receptors are acquired by NK cells during their maturation process, thus regulating their trafficking within the body (Walzer and Vivier, 2011). The quantity of NK cells depend upon the organ analyzed. NK cells can also acquire tissue-specific functions, as postulated for uterine NK cells during placentation process (Hanna et al., 2006).

Infiltrating cells expressing NK receptors (NKR) have been detected in tissues of patients suffering from diverse inflammatory disorders, prompting to investigate the role of NK cells in these situations (Schleinitz et al., 2010). However, quantitative and qualitative changes of NK cells that can be associated with disease conditions cannot be properly evaluated without a precise knowledge of NK cell tissue distribution at steady state. Currently, human NK cells have been mainly studied in lymphoid tissues, restricting the knowledge of their distribution in non-lymphoid tissues to extrapolation from studies in the mouse model (Shi et al., 2011). One of the limitations to extend these studies to humans resides in the poor availability of healthy human tissues, in particular non-lymphoid ones. Another major limitation to translation of findings obtained in mice to humans has been represented for a long time by the absence of markers conserved between mice and humans. Indeed, human NK cells express an array of either inhibitory or activating receptors undergoing high rate of evolution, even when compared to high-related chimpanzees (Parham et al., 2012), and that is quite different from that expressed by mouse NK cells (Vivier et al., 2008). The Natural Cytotoxicity Receptor (NCR) encompass three molecules initially described in human NK cells: NKp30, NKp44, and NKp46. While NKp44 expression is mainly restricted to primates, NKp30 is present also on rat NK cells (Hsieh et al., 2006) and NKp46 is evolutionary conserved in mammals, thus representing the best candidate to identify NK cells in various mammalian species (Walzer et al., 2007b). However, in mice NKp46 can be also found on rare T cell subsets (Stewart et al., 2007; Walzer et al., 2007a) as well as on some gut Innate Lymphoid Cells (ILCs) cell subsets expressing the retinoic acid receptor-related orphan receptor γt (RORγt) transcription factor (Satoh-Takayama et al., 2008; Luci et al., 2009; Sanos et al., 2009). We collected the samples of both lymphoid and non-lymphoid organs isolated from human brain-dead donors, and analyzed the distribution and the phenotype of NKp46^+^ cells in various healthy human tissues.

## Results

### Tissue distribution of NKp46^+^ cells in human healthy lymphoid and non-lymphoid tissues

**Figure 1:**
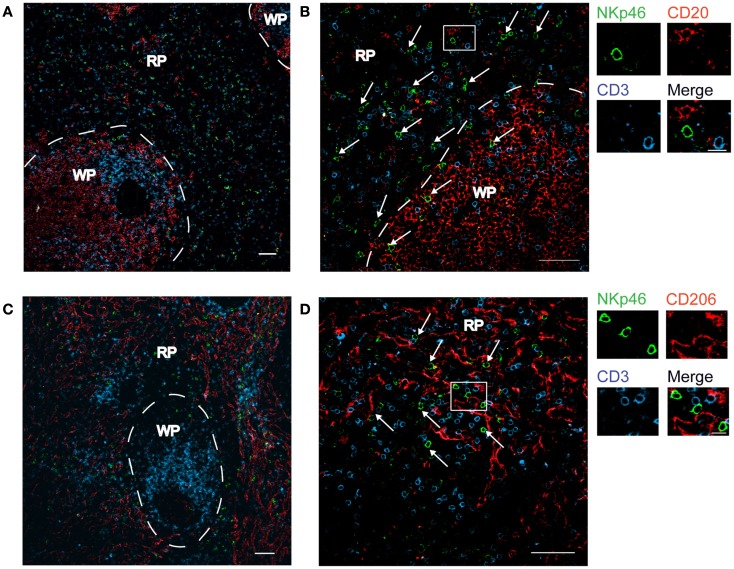
**Distribution of NKp46^+^ cells in the spleen**. **(A–D)** Frozen sections of human spleen were stained with polyclonal anti-NKp46 serum (green), anti-CD20 mAb (red), and anti-CD3 mAb (blue) **(A,B)** or with polyclonal anti-NKp46 serum (green), anti-CD206 mAb (red), and anti-CD3 mAb (blue) **(C,D)**. WP, white pulp; RP, red pulp. Arrows indicate NKp46^+^CD3^−^ cells. Scale bar = 50 μm. Data are representative of at least two independent experiments performed on three distinct individuals.

**Figure 2:**
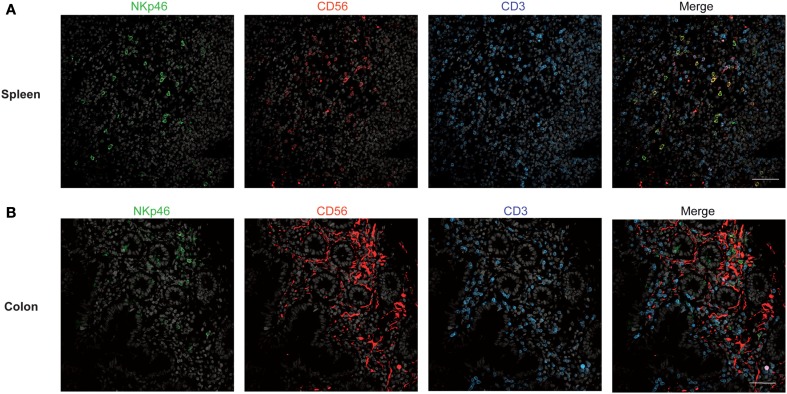
**Staining with anti-NKp46, anti-CD56, and anti-CD3 in human spleen and gut**. **(A,B)** Frozen sections of human spleen **(A)** or colon **(B)** were stained with polyclonal anti-NKp46 serum (green), anti-CD56 mAb (red), and anti-CD3 mAb (blue). Nuclei were counterstained with Sytox (gray). Scale bar = 50 μm. Data are representative of at least two independent experiments performed on three distinct individuals.

**Figure 3:**
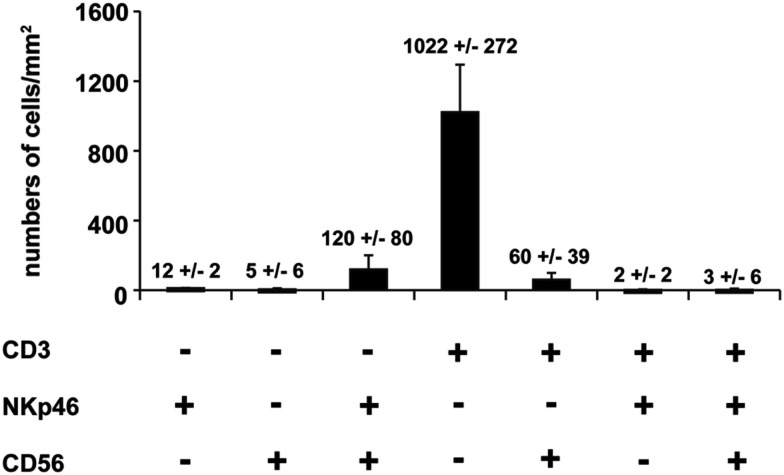
**Quantification of splenic cells expressing CD56, NKp46, or CD3**. Cells with indicated phenotypes were quantified on human spleen sections. Data (mean ± SEM) are representative of three distinct individuals. Corresponding values (mean ± SEM) are indicated at the top of the corresponding bars. Counting of the indicated cell subsets was performed in 2–5 distinct areas of tissue sections.

We first investigated the distribution of NKp46^+^ cells in tissue sections of various lymphoid and non-lymphoid organs prepared from healthy individuals. In the spleen, the splenic white pulp (WP) is constituted by the T cell zone and B-cell follicles surrounding splenic central arterioles (Mebius and Kraal, 2005). WP lymphoid structures are disseminated within the red pulp (RP), containing abundant macrophages or dendritic cells expressing the Macrophage Mannose Receptor-1/CD206 molecule and lining splenic venous sinuses (Pack et al., 2008). NKp46^+^ cells were rare within organized CD3^+^ T cell and CD20^+^ B-cell lymphoid zones (Figure 1). Most NKp46^+^ cells were present as scattered cells in CD206^+^ RP areas surrounding WP lymphoid structures (Figure 1). The large majority of CD3^−^NKp46^+^ cells were CD56^+^ (Figures 2A and 3). These findings show that human splenic NKp46^+^ cells mainly localize in the RP at steady state, as in mouse model (Gregoire et al., 2008). In this organ, the selective expression of NKp46 expression to NK cells was confirmed by the barely detectable presence of T cells expressing NKp46, which contrasts with the expression of CD56 on 2–8% of CD3^+^ cells (Figures 2A and 3).

**Figure 4:**
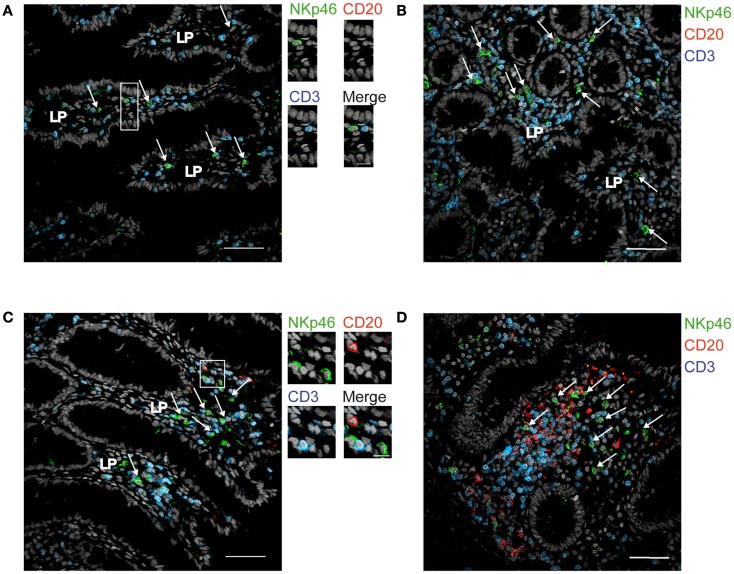
**Distribution of NKp46^+^ cells in the gut**. **(A,B)** Frozen sections of human ileum **(A,B)** or of human colon **(C,D)** were stained with polyclonal anti-NKp46 serum (green), anti-CD20 mAb (red), and anti-CD3 mAb (blue). Nuclei were counterstained with Sytox (gray). Arrows indicate NKp46^+^CD3^−^ cells. LP, lamina propria. Scale bar = 50 μm. Data are representative of at least two independent experiments performed on three distinct individuals.

**Figure 5:**
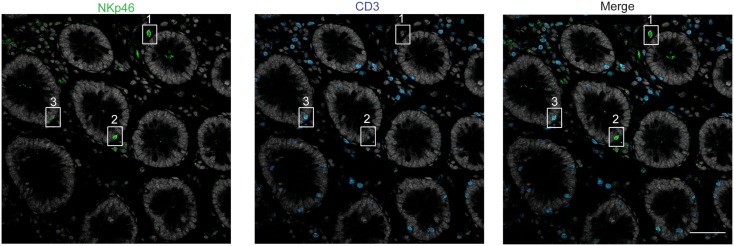
**Staining with anti-NKp46 and anti-CD3 on human colon**. Frozen sections of human colon were stained with polyclonal anti-NKp46 serum (green) and anti-CD3 mAb (blue). Nuclei were counterstained with Sytox (gray). Scale bar = 50 μm. Inset 1 = NKp46^+^CD3^−^ in gut lamina propria; inset 2 = NKp46^+^CD3^−^ within epithelial cell layer; inset 3 = NKp46^+^CD3^+^ within epithelial cell layer. One representative experiment performed on three distinct individuals.

We then analyzed the distribution of NKp46^+^ cells in human small and large intestine. Consistent with our previous findings (Luci et al., 2009), NKp46^+^ cells were mainly detectable within the lamina propria of ileum villi (Figures 4A,B) and of colonic crypts (Figures 4C and 5, inset 1). CD56, the Neural Cell Adhesion molecule (N-CAM) has been used since decades to identify NK cells in humans. In the gut, CD56 stained numerous CD45^−^ cells (data not shown), most likely corresponding to N-CAM^+^ nervous fibers (Driessen et al., 1995), and preventing the unambiguous identification of CD56^+^NKp46^+^ cells in the gut (Figure 2B). Within epithelial cell layer, we detected rare NKp46^+^CD3^−^ (Figure 5, inset 2) and NKp46^+^CD3^+^ cells (Figure 5, inset 3). NKp46^+^ cells were also found in colonic lymphoid aggregates enriched in T and B lymphocytes (Figure 4D). This localization was reminiscent of NKp46^+^ cells expressing the RORγt transcription factor and present in gut-associated lymphoid tissues in the mouse (Sanos et al., 2009; Vivier et al., 2009; Reynders et al., 2011).

### Expression of NKp46 on gut RORγt^+^ cells

**Figure 6:**
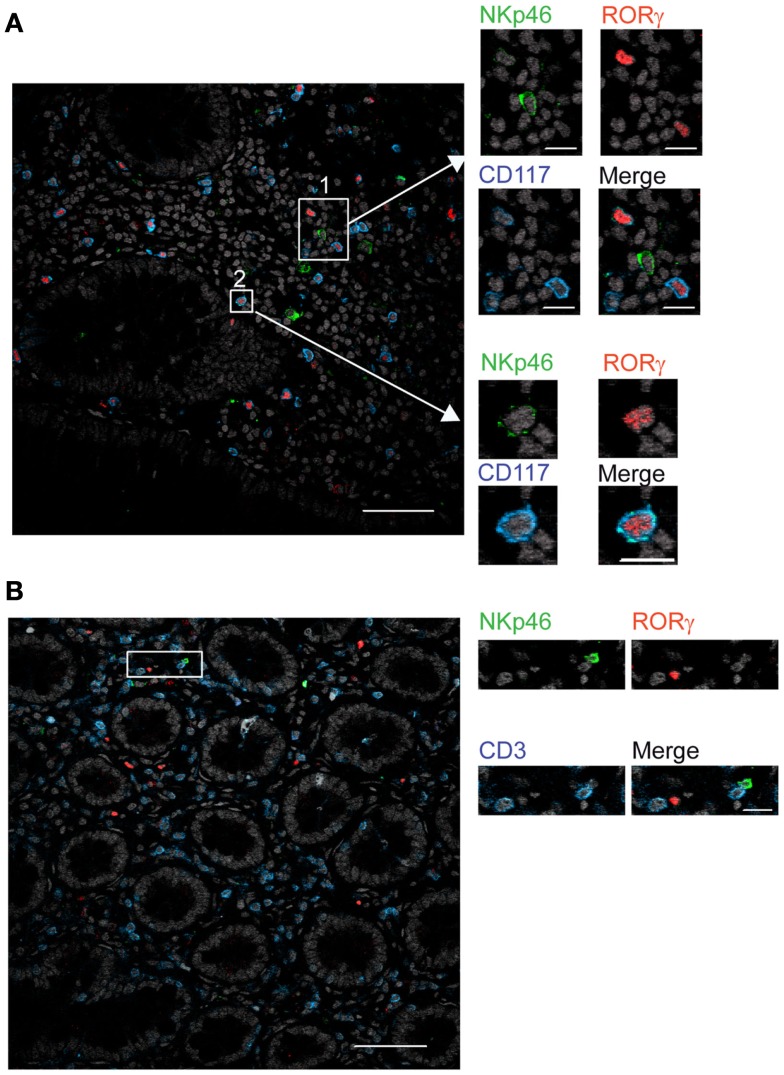
**RORγt^+^ cells in the gut express low cell surface density of NKp46**. **(A,B)** Frozen sections of human colon were stained with **(A)** polyclonal anti-NKp46 serum (green), anti-RORγt mAb (red), and anti-CD117 mAb (blue) or with **(B)** polyclonal anti-NKp46 serum (green), anti-RORγt mAb (red), and anti-CD3 mAb (blue). Nuclei were counterstained with Sytox (gray). Scale bar = 50 μm. Data are representative of at least three independent experiments performed on three distinct individuals.

In mouse gut-associated lymphoid tissues, the differential expression of the RORγt transcription factor discriminates two NKp46^+^ cell subsets: the NKp46^+^RORγt^+^, a subset of ILCs that produces IL-22, and conventional NKp46^+^RORγt^−^ cells (cNK cells), that produces IFN-γ (Satoh-Takayama et al., 2008; Luci et al., 2009; Sanos et al., 2009; Reynders et al., 2011). We thus investigated whether human gut NKp46^+^ expressed RORγt. Scarced RORγt^+^ cells were found in the gut lamina propria. These cells expressed the c-kit receptor, CD117 (Figure 6A) and were largely CD3^−^ (Figure 6B), consistent with their classification as RORγt^+^ILCs (Cupedo et al., 2009; Crellin et al., 2010). RORγt^+^CD117^+^ cells were found to be NKp46^−^ (Figure 6A, inset 1) or NKp46^low^ (Figure 6A, inset 2). Subsets of RORγt^+^ILCs present in human tonsils can express other NK cell markers, such as CD56 (Cupedo et al., 2009; Crellin et al., 2010). However, the use of this marker in histology was not applicable to intestinal tissues (Figure 2B). Our results thus indicate that, differently from the mouse model, NKp46 is barely detectable on human gut RORγt^+^CD117^+^ ILCs in histology. Thus, either NKp46^+^RORγt^+^ cells are not present in human, or their cell surface expression of NKp46 is too low to be unambiguously visualized in tissue sections. These data also imply that the vast majority of NKp46^+^CD3^−^ cells readily detected by immunohistology in gut lamina propria and lymphoid aggregates are cNK cells.

### Expression of CD56 and NKp46 in lymphoid and non-lymphoid tissues

**Figure 7:**
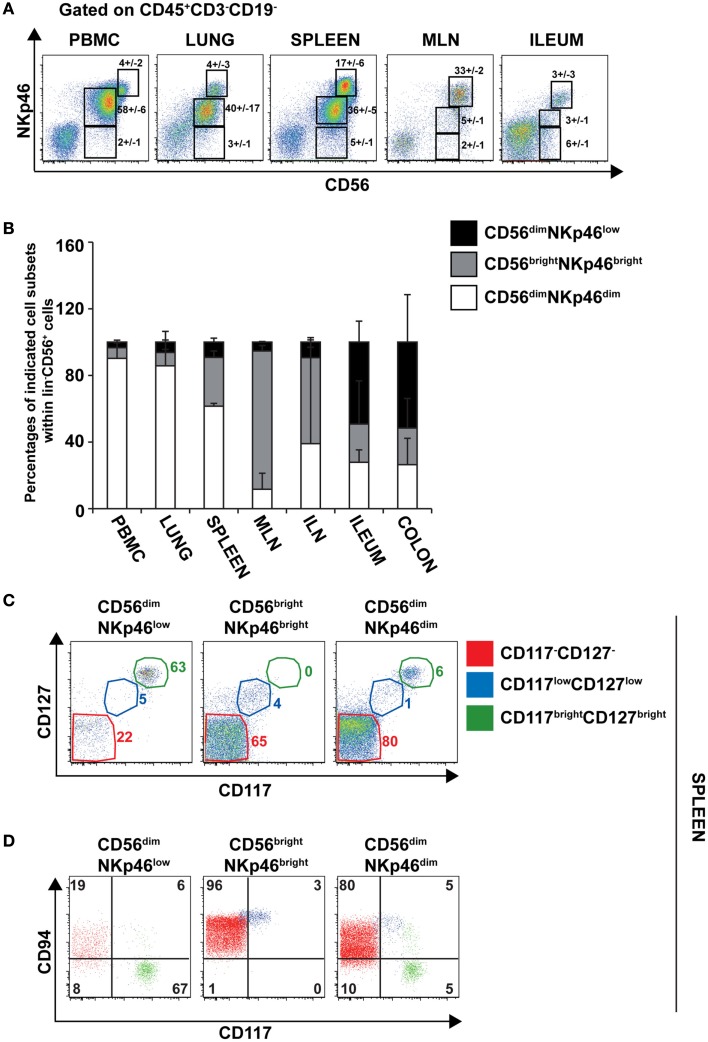
**Surface expression of CD56 and NKp46 identifies three distinct cell subsets in human lymphoid and non-lymphoid organs**. **(A)** Flow cytometry analysis of cells isolated from the indicated human organs, by gating on CD45^+^CD3^−^CD19^−^ cells. Numbers (mean ± SEM) in quadrants indicate percentages of cells. Data are representative of at least three independent experiments. **(B)** Proportions (mean ± SEM) of indicated cell subsets within CD56^+^CD45^+^CD3^−^CD19^−^ cells. *N* = 6–8 for PBMC, spleen, lung, ileum, and colon; *N* = 2–3 for ILN and MLN. **(C)** Flow cytometry analysis of cell surface expression of indicated cytokine receptors on splenic CD56^dim^NKp46^low^, CD56^bright^NKp46^bright^, and CD56^dim^NKp46^dim^ cell subsets. Data are representative of at least three independent experiments. Red gate, CD117^−^CD127^−^ cells; blue gate, CD117^low^CD127^low^ cells; green gate, CD117^bright^CD127^bright^ cells. **(D)** Flow cytometry analysis of CD94 and CD117 expression on indicated cell subsets of CD45^+^CD3^−^CD19^−^ splenocytes. Numbers in quadrants indicate percentages of cells. One experiment representative of three. Red cells were gated on CD117^−^CD127^−^ cells; blue cells were gated on CD117^low^CD127^low^ cells; green cells were gated on CD117^bright^CD127^bright^ cells.

To further document the expression of NKp46 in human tissues, we investigated by flow cytometry the distribution of NKp46^+^ cells in various lymphoid and non-lymphoid organs. NKp46 was analyzed with respect to CD56. Consistent with published data (Freud and Caligiuri, 2006), CD56^bright^NKp46^bright^ cells were abundant in secondary lymphoid organs, such as the mesenteric lymph nodes (MLN) and the iliac lymph nodes (ILN), while CD56^dim^NKp46^dim^ cells were predominant in the peripheral blood, the lung, and the spleen (Figures 7A,B). Similar proportions of these two cell subsets were detected in the gut (Figures 7A,B). Besides the well-known dichotomy between CD56^bright^ and CD56^dim^ NK cells, the use of NKp46 allowed us to identify CD56^dim^NKp46^low^ cells (Figure 7A). Within CD56^+^ cells this novel cell subset was abundant in both ileum and colon, but rare, although detectable, in the other lymphoid and non-lymphoid organs studied (Figures 7A,B).

**Figure 8:**
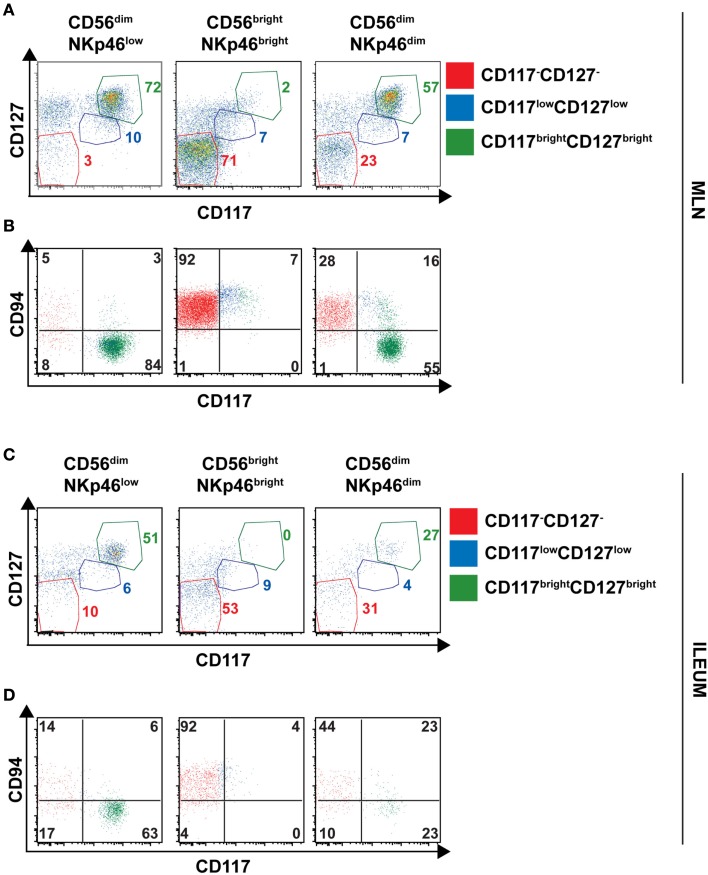
**Expression of CD127, CD117 and CD94 on CD56^+^ cell subsets of human lymph nodes and ileum**. **(A–D)** Flow cytometry analysis of cell surface expression of indicated molecules on cells isolated from indicated organs. Red gate, CD117^−^CD127^−^ cells; blue gate, CD117^low^CD127^low^ cells; green gate, CD117^bright^CD127^bright^ cells. **(A,B)**, Mesenteric lymph node cells; **(C,D)**, Ileum LPC.

The low NKp46 expression in CD56^dim^ cells was reminiscent of CD117^+^RORγt^+^NKp46^low^ ILCs detected in gut tissue sections (Figure 6A). In both human and mouse, RORγt^+^ILCs express high amounts of CD117 and CD127 (receptors for stem cell factor and IL-7, respectively; Vivier et al., 2009; Sawa et al., 2010), while in NK cells the expression of these cytokine receptors is restricted to cell precursors (Freud and Caligiuri, 2006; Crellin et al., 2010). In the spleen, lymph nodes, and gut, CD56^dim^NKp46^low^ cells enclosed up to 72% of CD117^bright^CD127^bright^ cells (Figures 7C and 8A,C). However, CD117^−^CD127^−^ cells could also be detected within CD56^dim^NKp46^low^ cell subset (Figure 7C). Within CD56^dim^NKp46^dim^ cell, CD117^bright^CD127^bright^, and CD117^−^CD127^−^ cell proportions varied depending on the organ analyzed, the latter being predominant in the spleen (Figures 7C and 8A,C). In contrast, CD56^bright^NKp46^bright^ were mainly CD117^−^CD127^−^ cells (up to 71% of cell subset) in all organs analyzed (Figures 7C and 8A,C). Finally, we detected also a minor fraction of CD117^low^CD127^low^ cells in CD56^dim^NKp46^low^, CD56^bright^NKp46^bright^, and CD56^dim^NKp46^dim^ cells subsets (Figures 7C and 8).

In NK cells, CD94 is present on both CD56^bright^ and CD56^dim^ cell subsets, but undetectable on earlier NK cell maturation stages, such as stage 1 (CD34^+^CD117^−^ cells), 2 (CD34^+^CD117^+^ cells), and 3 (CD34^−^CD117^+^ cells; Freud et al., 2005). We then investigated the expression of CD94 with respect to CD117 in CD56^dim^NKp46^low^, CD56^bright^NKp46^bright^, and CD56^dim^NKp46^dim^ cells subsets. As expected, CD94 was expressed on CD117^−^ cells (Figures 7D and 8B,D, red gate), but absent on CD117^bright^ cells of all the three subsets analyzed (Figures 7D and 8B,D, green gate). Yet, CD94 was expressed on CD117^low^CD127^low^ cell subset (Figures 7C and 8, blue gate).

In conclusion, our results highlight an heterogeneity of human CD56^dim^ cells expressing low (NKp46^low^) to intermediate (NKp46^dim^) amounts of NKp46 at their cell surface. These heterogeneous populations include CD117^bright^CD127^bright^CD94^−^ cells and CD117^−^CD127^−^CD94^+^ cells, phenotypically reminiscent of RORγt^+^ILC and cNK cells, respectively.

### Characterization of splenic cNK cell and RORγt^+^ ILC cell subsets

**Figure 9:**
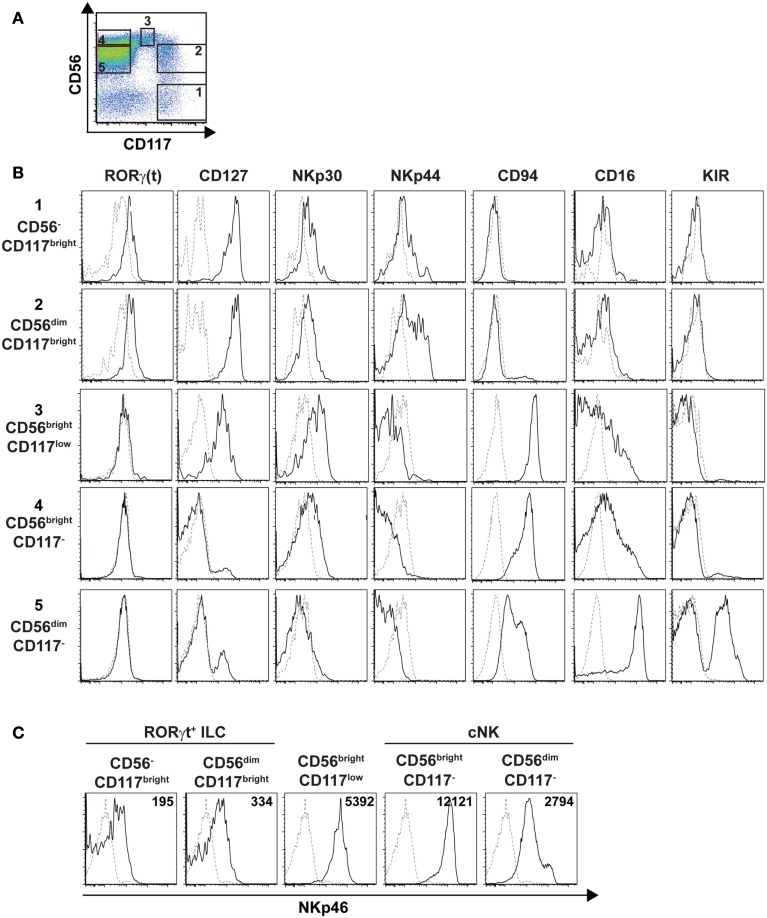
**Phenotypic profiles of splenic cNK and RORγt^+^ILC cell subsets**. **(A)** Flow cytometry analysis of CD56 vs. CD117 expression on splenic human cells, by gating on CD45^+^CD3^−^CD19^−^ cells. Data are representative of at least three independent experiments. Numbers indicate the following cell subsets: (1) CD56^−^CD117^bright^ cells; (2) CD56^dim^CD117^bright^ cells; (3) CD56^bright^CD117^low^ cells; (4) CD56^bright^CD117^−^ cells; (5) CD56^dim^CD117^−^ cells. **(B,C)** Expression of the indicated ILC or NK cell markers **(B)** or of the NKp46 cell marker **(C)** on indicated splenic cell subsets defined as in **(A)**. Black line, specific staining; dashed line, isotype control staining in **(B)** or staining on CD56^−^CD117^−^ cells in **(C)**. Numbers in **(C)** represent the mean fluorescence intensity of NKp46 expression in indicated cell subsets.

As our results showed that the cell surface density of NKp46 varies between human RORγt^+^ ILC and cNK, we decided to pursue our phenotypic analysis by comparing well-defined subsets of these two cell lineages for their cell surface expression of NKp46. In humans cNK were defined as lineage^−^CD56^+^CD117^−^CD127^−^RORγt^−^ cells, while adult RORγt^+^ ILCs, enclosing Lymphoid Tissue inducers (LTi)-like cells, were characterized as lineage^−^CD117^bright^CD127^bright^RORγt^+^ cells and included a CD56^+^ cell fraction (Cupedo et al., 2009; Crellin et al., 2010). By combining CD56 and CD117 staining, we discriminated within CD45^+^CD3^−^CD19^−^ splenic cells the following five populations: (1) CD56^−^CD117^bright^ cells; (2) CD56^dim^CD117^bright^ cells; (3) CD56^bright^CD117^low^ cells; (4) CD56^bright^CD117^−^ cells; (5) CD56^dim^CD117^−^cells (Figure 9A). CD56^−^CD117^bright^ and CD56^dim^CD117^bright^ cells were RORγt^+^CD127^bright^NKp30^+^, but they do not express NKR such as CD94, CD16, and KIR. The NCR NKp44 was expressed on a fraction of CD56^dim^CD117^bright^ cells (Figure 9B). In contrast, CD56^bright^CD117^−^ and CD56^dim^CD117^−^cells were mainly RORγt^−^CD127^−^ NKp30^low^NKp44^−^ and expressed CD94, CD16, and KIR according to the well-known phenotypic profiles of peripheral CD56^bright^ and CD56^dim^ cNK cell subsets, respectively (Figure 9B). CD56^bright^CD117^low^ were RORγt^−^CD127^low^ and expressed a phenotypic profile similar to that of CD56^bright^CD117^−^ cells for the NKp30, NKp44, CD94, CD16, and KIR markers (Figure 9B).

We then analyzed the cell surface expression of NKp46 on splenic RORγt^+^ ILCs and cNK cell subsets. Consistent with our previous findings (Figures 7 and 8), NKp46 was detectable at low levels on both CD56^−^ and CD56^dim^ fractions of CD117^bright^ cells (Figure 9C). In contrast, NKp46 was expressed at high levels on CD56^bright^CD117^−^ cells and CD56^dim^CD117^−^ cells, as well as on CD56^bright^CD117^low^ cells (Figure 9C). Nevertheless, a minor fraction of NKp46^low^ cells was detectable in all these three cell subsets (Figure 9C). Altogether, these data confirm that NKp46 is poorly expressed on RORγt^+^ ILCs, while it stains brightly most of cNK cells.

### Characterization of gut cNK cell and RORγt^+^ ILC cell subsets

To further characterize RORγt^+^ ILC cell and cNK cell subsets present in the gut, we performed genome-wide expression profiling of sorted CD56^−^CD117^bright^CD127^bright^ cells and CD56^+^CD117^−^CD127^−^ cells (thus containing both CD56^bright^CD117^−^ and CD56^dim^CD117^−^ cells) isolated from human colon. Genes upregulated at least twofold in CD56^−^CD117^bright^CD127^bright^ cells with respect to CD56^+^CD117^−^CD127^−^ cells included *IL-22*, *KIT*, *TNFSF11*, *IL1R1*, *RORC*, and *IL7R*, thus encoding molecules well-known as involved in RORγt^+^ ILC development and function (Table 1; Table 2 in Supplementary Material). In contrast, genes upregulated at least twofold in CD56^+^CD117^−^CD127^−^ with respect to CD56^−^CD117^bright^CD127^bright^ cells encompassed *GZMA*, *GZMK*, *GZMH*, *IFNG*, *CCL4*, *CCL5*, encoding key molecules regulating NK cell cytotoxicity and cytokine secretion (Table 1; Table 3 in Supplementary Material). Phenotypic analysis in the spleen and genome-wide profiling in the gut thus allowed us to unequivocally assign CD56^−^CD117^bright^ and CD56^dim^CD117^bright^ to the RORγt^+^ ILCs and CD56^bright^CD117^−^ cells and CD56^dim^CD117^−^ cells to cNK cells.

**Table 1:** **Example of genes upregulated at least twofold in the reciprocal comparison between CD56^−^CD117^bright^CD127^bright^ and CD56^+^CD117^−^CD127^−^ cells**.

Representative genes upregulated in CD56^−^ CD117^bright^CD127^bright^ with respect to CD56^+^CD117^−^ CD127^−^ cells	Fold change	Representative genes upregulated in CD56^+^CD117^−^CD127^−^ with respect to CD56^−^CD117^bright^ CD127^bright^ cells	Fold change
*IL-22*	10,8	*GNLY*	4,8
*KIT*	3,1	*GZMA*	3,9
*TNFSF11*	3,0	*IFNG*	3,9
*IL1R1*	2,9	*GZMK*	3,9
*TNFRSF11A*	2,7	*CCL4*	3,6
*RORC*	2,6	*EOMES*	3,1
*TNFSF4*	2,4	*GZMH*	2,6
*IL7R*	2,3	*CCL5*	2,4

## Discussion

The characterization of NK cells according to their anatomical localization is paramount to unveil the physiological role of these unconventional lymphocytes. By using the NKp46 NK cell marker, we reported here an extensive analysis of NKp46^+^ cell localization, frequency, and phenotype in lymphoid and non-lymphoid tissues isolated from human healthy donors.

Our work shows that the use of NKp46 for the identification of human cNK cells presents important advantages with respect to other NK cell markers, such as CD56. Indeed, in gut sections CD56 staining on intestinal neural fibers prevented proper detection of hematopoietic CD3^−^CD56^+^ cells; hence NK cells could be identified as CD3^−^NKp46^+^RORγt^−^ cells. Moreover, with respect to T lymphocytes, CD3^+^CD56^+^ cells were at least 30-times more abundant than the few detectable CD3^+^NKp46^+^ cells in all organs analyzed (Figure 3 and data not shown), thus confirming that NKp46^+^ cells mainly consist of cNK cells. Altogether, our findings indicate that, as already shown in other mammalian species (Storset et al., 2004; Walzer et al., 2007b; Jozaki et al., 2010; Connelley et al., 2011), the expression of NKp46 is a reliable tool to identify cNK in human healthy tissues in histology and flow cytometry. These expression data thus establish the basis for analyzing quantitative and qualitative changes of NK cell compartment that could be associated with various clinical conditions in humans.

Our analysis also revealed that, besides the well-characterized CD56^bright^NKp46^bright^ and CD56^dim^NKp46^dim^ subsets of cNK cells, CD56^dim^ cells include cells with low expression of NKp46 phenotypically related to the RORγt^+^ ILCs (CD56^dim^NKp46^low^CD117^bright^CD127^bright^CD94^−^) or to the NK cell lineage (CD56^dim^NKp46^low^CD117^−^CD127^−^CD94^+^). CD56^dim^NKp46^low^CD117^bright^CD127^bright^CD94^−^ cells expressed RORγt. When isolated from gut, but not from spleen, these cells were able to secrete IL-22 upon IL-23 stimulation (data not shown). Accordingly, a RORγt^+^ ILC signature, including genes such as *RORC*, *IL-22*, *IL1R1*, *IL7R*, *KIT* and conserved since fetal life in mice (Reynders et al., 2011) was detected in CD117^bright^CD127^bright^ cells isolated from human colon (Table 1; Table 2 in Supplementary Material). Altogether, these findings support the classification of CD117^bright^CD127^bright^CD94^−^ cells within CD56^dim^NKp46^low^ cells as RORγt^+^ ILCs. Consistent with our data, RORγt^+^ILCs present in human tonsils, lymph nodes, and gut harbor low NKp46 cell surface expression (Cella et al., 2009; Cupedo et al., 2009; Crellin et al., 2010; Takayama et al., 2010). In contrast, the CD117^−^CD127^−^CD94^+^ cells identified within CD56^dim^NKp46^low^ cells remain poorly characterized. Their cell surface phenotype is rather consistent with their belonging to the NK cell lineage. However, their position along NK cell maturation stages is unclear. They could represent an immature CD94^+^ NK cell subset harboring low amounts of NKp46. Alternatively, they could include NK cells that downregulated NKp46 upon receptor engagement. Further molecular and developmental studies are required to disentangle their origin and function within NK cell lineage.

Differently from what shown in mice (Satoh-Takayama et al., 2008; Luci et al., 2009; Sanos et al., 2009), NKp46 cannot be used by itself to identify NKR^+^ subsets of human RORγt^+^ILCs. These differences could be related to both evolutionary and environmental factors. Indeed, NKp46 is the only NCR expressed in murine cells, while human cells can express other NCRs such as NKp44 and NKp30. NKp30 was detectable, although at low levels, on both NK and RORγt^+^ILCs, while NKp44 was found on a large fraction of RORγt^+^ILCs, consistent with previous reports (Cella et al., 2009; Cupedo et al., 2009; Crellin et al., 2010; Takayama et al., 2010). Distinct NCRs could regulate RORγt^+^ILC functions in different mammalian species. In mice, NKp46 is dispensable for IL-22 production by RORγt^+^ILC (Satoh-Takayama et al., 2009). Indeed, IL-22 production is detectable in RORγt^+^ILC since fetal life, early before the acquisition of NKp46 expression, and is regulated by IL-1-dependent, but commensal flora-independent mechanisms (Reynders et al., 2011). It cannot be excluded that NCRs could be involved in other RORγt^+^ILC functions. Adult RORγt^+^ILC can regulate CD4^+^ T cell memory responses (Lane et al., 2012). In NK cells, NKp46 participates to the tuning of effector function (Narni-Mancinelli et al., 2012). Whether NKp46 or NKp44 are involved in these functions in NKR^+^RORγt^+^ILC remains to be investigated, as well as the NKp46 and NKp44 cellular ligands which remain to be identified to fully understand the biology of these receptors on all NCR^+^ cells.

## Materials and Methods

### Human tissues

All organ samples were obtained from cadaveric heart-beating donors at the end of the procedure of graft harvesting. According to the French law (articles L. 1232-3, L. 1241-5 et L. 1241-6 du code de la santé publique), all the procurements of samples were done in accordance with the rules of the Agency of the Biomedicine in France (Agence de la biomédecine), which approved and was informed of the entire experimental protocol (Protocol *N*° = PFS08-004). The Agency also verified the absence of opposition to donation of tissues for scientific issues, from the donor (registry) or his/her family. All samples were stored in cold phosphate buffer (PB) immediately after procurement. No information about the donor identity was available. All clinical and biological tools were accessible thanks to the donor chart available at the Agency of the Biomedicine.

Colon fragments used for extraction of intestinal cell subsets used for microarrays were obtained from patients affected by colon cancer that underwent surgical resection of segments of intestine. The biological specimens of colon used for the present study were “free” of any diseases, as assessed by the Unit of Pathology of the Istituto Clinico Humanitas, Milan, Italy. In particular, our pathologists evaluated the following histological features within the resection margins: edema, inflammation, lymphoid aggregates, pyloric metaplasia, fibrosis, cryptitis and crypt abscesses, ulcers, granulomas, villous shortening, mucin depletion, neuronal hyperplasia, and transmural inflammation, cancer infiltrates. Gut specimens with such characteristics were excluded from our study. Gut tissue specimens were obtained in accordance with clinical protocols approved by the Institutional Review Board (IRB) of Istituto Clinico Humanitas, Milan, Italy. Each patient signed a consent form that was approved by the above IRB that specified that the donation of gut specimens for this research project did not affect in any way the diagnosis, the therapies and the prognosis of the disease.

### Immunofluorescence (histology)

Human spleen, small and large intestine portions were isolated from human donors, extensively washed in PB, and then fixed in PB supplemented with 4% paraformaldehyde (PFA, Electron Microscopy Sciences, UK) for 2 h.

After three washes in PB, PFA-fixed organs were incubated overnight in a PB supplemented with 30% sucrose. Tissues were then embedded in optimum cutting temperature compound (Sakura Finetek), frozen in a bath of isopentane cooled on dry ice, and cut in 8 μm-thick sections. Human NKp46 staining was then performed using polyclonal goat anti-human NKp46 (R&D Systems, France), followed by Alexa488-conjugated donkey anti-goat antibodies (Molecular Probes, France). Human CD20 staining was performed using monoclonal mouse anti-human (H1, BD Pharmingen, France), followed by Alexa555-conjugated donkey anti-mouse antibodies (Molecular Probes, France). Human CD3ε staining was performed using mouse anti-human Alexa647-conjugated mAb (BD Pharmingen, France). Goat IgG (R&D Systems, France) was used as isotype control for human NKp46 staining. Human CD206 staining was performed using monoclonal mouse anti-human (19.2, BD Pharmingen, France) followed by Alexa555-conjugated donkey anti-mouse antibodies (Molecular Probes, France). Human CD56 staining was performed using monoclonal mouse anti-human (123C3, Abcam) followed by Alexa488-conjugated donkey anti-mouse antibodies (Jackson Immunoresearch) or Alexa555-conjugated donkey anti-mouse antibodies (Molecular Probes, France). Human RORγt staining was performed using monoclonal rat anti-mouse/human RORγt (AFKJS-9, eBiosciences) followed by Cy3-conjugated donkey anti-rat antibodies (Jackson Immunoresearch). Human CD117 staining was performed using polyclonal rabbit anti-human CD117 (Dako) followed by Alexa647-conjugated donkey anti-rabbit antibodies (Jackson Immunoresearch). Purified mouse IgG2a isotype control was used as isotype control for CD20 staining and Alexa fluor 647-conjugated mouse IgG1 was used as isotype control for CD3. Purified mouse IgG1 (BD Pharmingen) was used as isotype control for CD206 and CD56 staining. Purified rat IgG2a (BD Pharmingen) was used as isotype control for RORγt staining. Rabbit IgG (R&D Systems, France) was used as isotype control for CD117 staining. Nuclear counterstaining was performed with Sytox (Invitrogen, France).

After staining, slides were dried, mounted with Prolong Gold (Invitrogen, France) and examined under Zeiss LSM 510 confocal microscope (Zeiss). Image processing was performed with Zeiss LSM and Adobe Photoshop softwares.

### Cell preparation and stimulation

Peripheral Blood Mononuclear Cells (PBMC) were enriched from total peripheral blood upon Ficoll gradient. Spleens were mechanically disrupted, then splenocytes were enriched upon Ficoll gradient. MLN and ILN cells were obtained after mechanical disruption. For preparation of cells from ileum and colon fragments, first we removed muscularis mucosae, then fragments were carefully washed in PBS 1×. After washing, gut pieces were cut into fragments of 2 cm-length and treated 3–4 times with PBS containing 5 mM EDTA (Gibco, France), 15 mM HEPES (Gibco, France), and 10% FCS at 37°C at 200 rpm for 20 min, in order to remove intraepithelial cells (IEC). After IEC extraction, intestinal pieces were washed once in RPMI 10% FCS, then minced. Lamina propria cells (LPC) were obtained by incubating three times the intestinal pieces with RPMI 10% FCS 15 mM HEPES (Gibco, France) containing 300 UI/ml of collagenase type VIII (Sigma Aldrich, France) at 37°C at 200 rpm for 15 min. LPCs were then enriched upon Percoll (Amersham-Pharmacia, France) gradient centrifugation. Lung fragments were minced, then digested as for gut LPC obtention. At the end of enzymatic digestions, cells were enriched upon Ficoll gradient.

### Flow cytometry

Cells were incubated with normal mouse serum (2 μl/sample) in the presence of different combination of following mAbs: FITC-conjugated anti-CD94 or anti-CD16 (both from BD Biosciences); PE-conjugated anti-NKp46, anti-NKp44 or anti-NKp30, or a mix of PE-conjugated anti-CD158a,h, anti-CD158b,j, and anti-CD158e1,e2 (all from Beckman Coulter); PerCP-Cy5.5-conjugated anti-CD3 (BD Biosciences) and PerCP-Cy5.5-conjugated anti-CD19 (Biolegend), PE-Cy7-conjugated anti-CD117 (Beckman Coulter), APC-conjugated CD56 (Beckman Coulter), Alexa700-conjugated anti-CD45 (Biolegend), and eFluor450-conjugated anti-CD127 (eBiosciences). FITC-conjugated, PE-conjugated and PE-Cy7-conjugated isotype-matched controls were from Beckman Coulter or BD Biosciences, while e450Fluor-conjugated isotype-matched control was from eBiosciences. After 30 min of incubation at 4°C, samples were washed once and fixed 10 min in PBS 1× containing 2% of paraformaldehyde. Samples were then washed in PBS 1×, resuspended in PBS 1× BSA 0.5%. Intracellular staining with anti-mouse/human RORγt-PE (clone AFKJS-9, eBioscience, France) was performed using the Foxp3 Fixation/Permeabilization kit (eBioscience, France), according to the manufacturer indications. Samples were run either on FACS Canto II or on LSRII cytometers (BD Biosciences, France) and analyzed by using Flow Jo 9.0.2 software (Tree Star Inc.).

### Microarray analysis

Lamina propria cells were isolated from macroscopically unaffected areas of colon of patients with colon cancer. Colonic CD56^−^CD117^bright^CD127^bright^ cells and CD56^+^CD117^−^CD127^−^ cells were sorted and immediately lysed in RLT buffer supplemented with 10% β-mercaptoethanol (Quiagen, France). Lysates of three distinct donors were pooled and RNA was isolated by using RNAeasy Microkit (Quiagen, France). Duplicates were performed for each cell type. cRNA were obtained after double amplification using the MessageAmp II aRNA Amplification Kit (Ambion, France). cRNA were then hybridized on Human Genome HG_U133 +2.0 Affymetrix chips. Chip images were generated using Affymetrix AGCC 3.2 software, then expression data were extracted and normalized using Affymetrix Expression console 1.1 with the algorithm RMA. Data obtained were expressed as log_2_. Raw and normalized data have been deposited in the GEO Database under reference number GSE41469. We selected 12278 probesets out of 54675 as “present,” when the probeset signal for at least one out of four samples was ≥ 6.5. For genes with multiple probesets, we selected the probeset giving the highest signal (8027 probesets out of 12278). Finally, we selected the probesets having linear fold changes ≥ 2 in all possible replicate comparisons between the two cell subsets analyzed.

## Conflict of Interest Statement

All authors concur with the submission of the manuscript and none of the data have been previously reported or are under consideration for publication elsewhere. Eric Vivier is cofounder and shareholder of Innate Pharma. The other authors declare no conflict of interest.

## Supplementary Material

The Supplementary Material for this article can be found online at: http://www.frontiersin.org/NK_Cell_Biology/10.3389/fimmu.2012.00344/abstract

Supplementary Tables 2 and 3 **Lists of genes upregulated in comparison between cNK vs. ROR ILC**. Gene represented were upregulated at least twofold in CD56^−^CD117^bright^CD127^bright^ cells with respect to CD56^+^CD117^−^CD127^−^ cells (Table 2) or in CD56^+^CD117^−^CD127^−^ cells with respect to CD56^−^CD117^bright^CD127^bright^ cells (Table 3). Probeset ID, gene symbol, fold change values, representative public ID, and extensive gene titles are indicated for each selected probeset.
